# Outbreaks of COVID-19 among healthcare personnel in a U. S. veterans administration health care system site, June and August 2023

**DOI:** 10.1186/s12995-025-00474-5

**Published:** 2025-08-29

**Authors:** Ignacio A. Santana, Max Blumberg, Noreen Chan, Margaret J. Ansay, Phyllis C. Tien, Paul D. Blanc, Sandeep Guntur

**Affiliations:** 1 Division of Occupational, Environmental and Climate Medicine, University of California, San Francisco, CA USA; 2 Department of Medicine San Francisco Veterans Affairs Health Care System, Division of Occupational Medicine, San Francisco, CA USA; 3 Department of Medicine, Division of Infectious Diseases, San Francisco Veterans Affairs Health Care System, University of California, San Francisco, San Francisco, CA USA

**Keywords:** COVID-19, SARS-CoV-2, Masking, Outbreak, Occupational, Work-related infection, Employee vaccination, Ventilation

## Abstract

**Background:**

Occupational COVID-19 remains a challenge among healthcare personnel (HCP).

**Methods:**

This study documents three COVID-19 outbreaks that occurred among vaccinated HCP within a single health care system (HCS) in California, USA in June and August 2023. The Employee Health (EH) unit for the HCS conducted surveillance with structured interviews, identifying outbreaks in real-time.

**Results:**

In June, 10 of 25 staff (40%) at a rural outpatient clinic that serves the HCS contracted COVID-19. One week later, 10 of 90 staff (11%) at a second outpatient clinic were infected, with half the cases were staff of a single close-knit team. In August, a team-building retreat of staff from the main hospital of the HCS resulted in 15 of 23 participants (65%) contracting COVID-19. The combined attack rate for these outbreaks was 25% (95% CI: 18–32%).

**Discussion:**

These outbreaks, despite high vaccination rates among the employees, reveal gaps in infection control, underscoring the need for stricter masking, improved ventilation, and rigorous surveillance testing. Rapid response to outbreaks and reinforcing education on symptom-based work exclusions are critical to preventing transmission among health care workers (HCWs) and their patients.

**Supplementary Information:**

The online version contains supplementary material available at 10.1186/s12995-025-00474-5.

## Introduction

Work-acquired COVID-19 among HCP remains an ongoing occupational health challenge for health care delivery systems, despite high vaccination rates within the workforce. Nosocomial outbreaks that also infect HCP have been well documented [1–4]. Worker to worker outbreaks spread in non-health care environments has also been investigated [5–9]. Despite this, narrative reports of discrete clinic or hospital-based COVID-19 outbreaks among HCP independent of nosocomial outbreaks have not been well documented, even though such data are highly relevant to preventive actions. In June and August 2023, a large HCS based in northern California (CA), USA experienced three substantial occupational COVID-19 outbreaks among its HCP. We report the details of the investigations and responses to these three outbreaks, highlighting the importance of occupational health vigilance for COVID-19 even among vaccinated HCP in the background of high transmissibility of COVID-19 [3]. 

## Methods

Cases of COVID-19 among HCP were subject to ongoing surveillance by the EH unit of the San Francisco Veterans Affairs Health Care System (SFVAHCS). All cases underwent a structured intake interview by trained nursing staff. A detailed intake was completed through data entry by a team of trained health care providers using a COVID-19 intake form custom-designed on a Microsoft Office Teams web application. Pertinent questions included: job title; place of work; HCS service or department; the reason for the call to the dedicated hotline; symptoms of COVID-19; date of symptom onset; whether the caller had any unmasked encounters with any employees within two days prior to the onset of symptoms (for contact tracing); whether appropriate personal protective equipment (PPE) had been used; whether there had been any recent household exposure to COVID-19; whether or not any household members were currently COVID-19 positive; and any recent travel outside of the US. The full intake questionnaire is provided as Appendix I.

There were 46 questions on the intake questionnaire as shown in Appendix I. The duration of the telephone-based intake interview ranged from 20 to 40 min. Routinely, there were also one or two follow-up telephone calls per case. We defined an outbreak as being three or more staff with a diagnosis of COVID-19 that was epidemiologically linked in space and time. Genotyping was not performed. Outbreaks were identified in real time as they unfolded based on the data reported by HCP.

After approval from SFVAHCS Executive Leadership Team (ELT), we undertook site visits site visits for Outbreaks 1 and 2 by one of the authors (MB) to collect additional data to garner additional details for these two outbreaks. These site visits included a tour of each facility and discussion with the clinic director and/or nurse manager, as well as speaking with a few of the staff who had contracted COVID-19 during the outbreak (after their return to work and following verbal consent). The duration of each site visit was approximately 1.5 hours. A final report was submitted to the ELT with the recommendations to prevent future outbreaks. Outbreak 3 cases were identified and reviewed by one of the authors (NC) based on the data from the SFVAHCS COVID-19 telephone hotline tracker.

## Results

### Overview

We interviewed a total of 138 persons, of which there 35 confirmed COVID-19 cases over three separate outbreaks. The overall attack rate was 25% (95% Confidence Interval [CI] 17.8 to 32.2%). The attack rates by outbreak and the occupational professions of the cases are presented in Table 1.

**Table 1:** Work-Related outbreaks case description

Outbreak	Attack Rate (95% CI)	Total Staff	Total Cases	Cases (Category)					
				Clinical Providers	Nursing	Social Worker	Medical Support Staff	Environmental Material Services	Other
1	40% [25.5, 64.5]	25	10	2	3	1	1	2	1(Telehealth through spouse)
2	11% [4.5, 17.5]	90	10	2	4	None	2	None	1(Lab Technician), 1(Security Guard)
3	65% [45.5, 84.5]	23	15	None	15	None	None	None	None
Combined	25% [17.8, 32.2]	138	35	4	22	1	3	2	3

### Outbreak 1

**Fig. 1:**
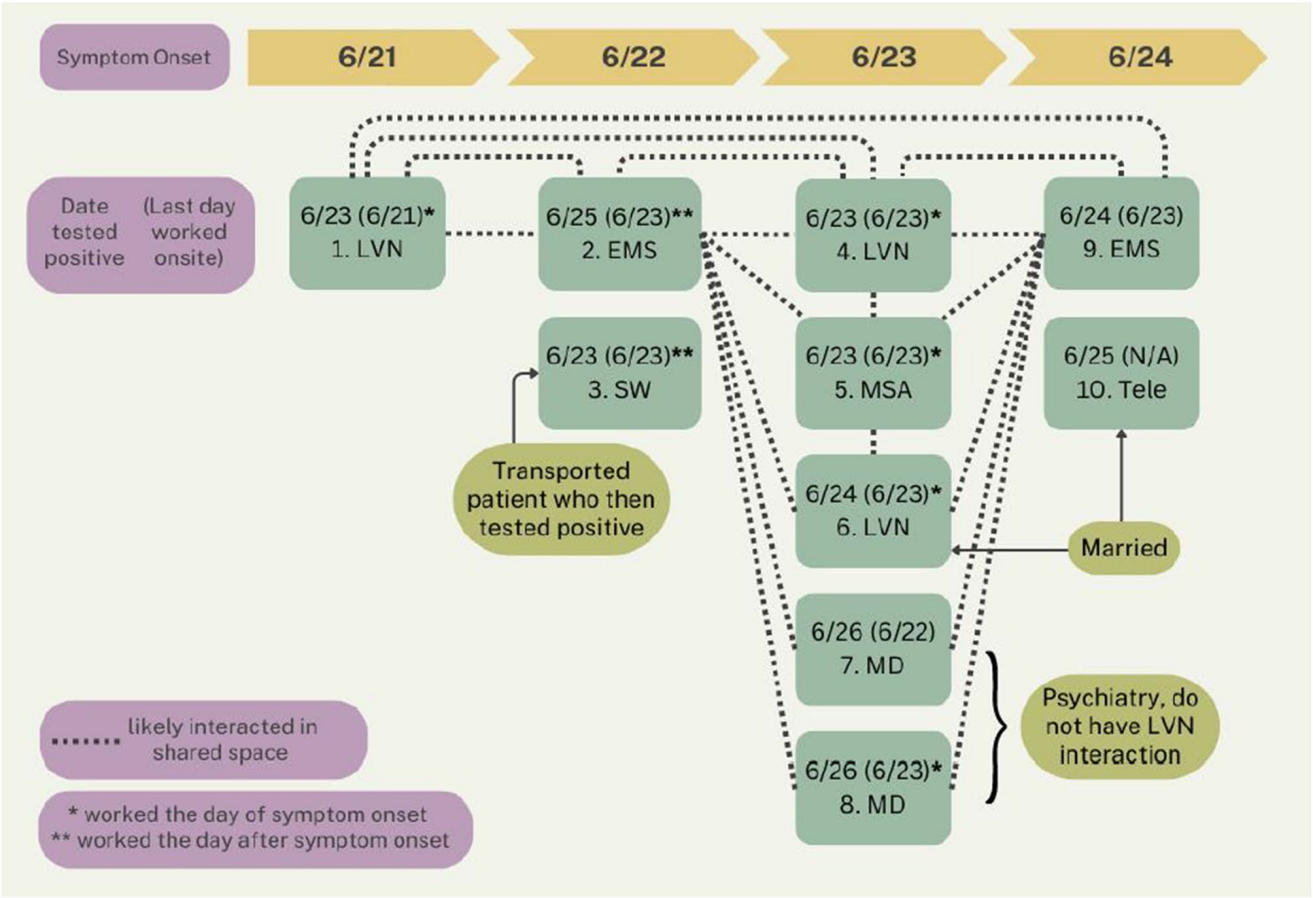
Outbreak 1. LVN = licensed vocational nurse; EMS = environmental management services; SW = social worker; MSA = medical support assistant; MD = physician; Tele = telehealth

In June 2023, a COVID-19 outbreak occurred in an SFVAHCS Community-Based Outpatient Clinic (CBOC) located in Clear Lake, California, USA. The outbreak is summarized schematically in Fig. 1. The clinic, with a staff of 25, is in a free-standing, leased building. It has a largely open floor plan for all staff operations outside of examination rooms, purportedly adequate but non-quantified air flow exchanges, and a mandatory masking policy only when engaged in direct patient care. Ultimately, 10 of 25 staff (40%) contracted COVID-19 in this outbreak.

### Outbreak 2

**Fig. 2:**
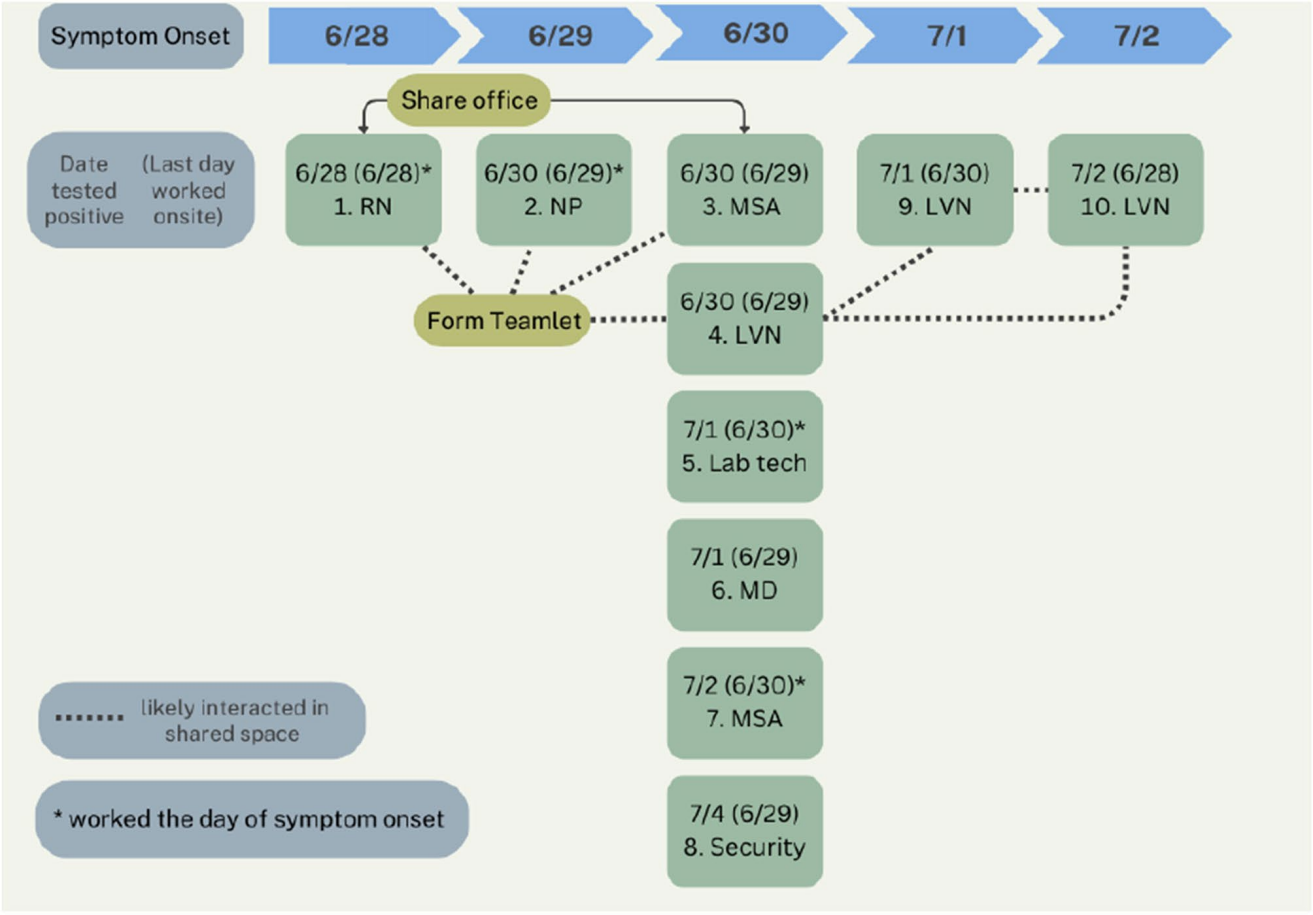
Outbreak 2. RN = registered nurse; NP = nurse practitioner; MSA = medical support assistant; MD = physician; LVN = licensed vocational nurse. “Form Teamlet” refers to the RN, NP, LVNs, and MSA that together worked as one teamlet

One week after Outbreak 1, a second occurred at another SFVAHCS CBOC, this one located in Eureka, California, USA. The outbreak is summarized schematically in Fig. 2. The clinic, with a staff of 90, also is in a free-standing leased building and has a largely open floorplan for staff operations outside of examination rooms, non-quantified air flow exchanges, and a mandatory masking policy only when engaged in direct patient care. Clinical staff were organized in cross-disciplinary “teamlets” with close physical interactions. Of 90 staff, 10 staff (11%) contracted COVID-19 within one week. Half of the new cases were from one teamlet of 5 members.

### Outbreak 3

**Fig. 3:**
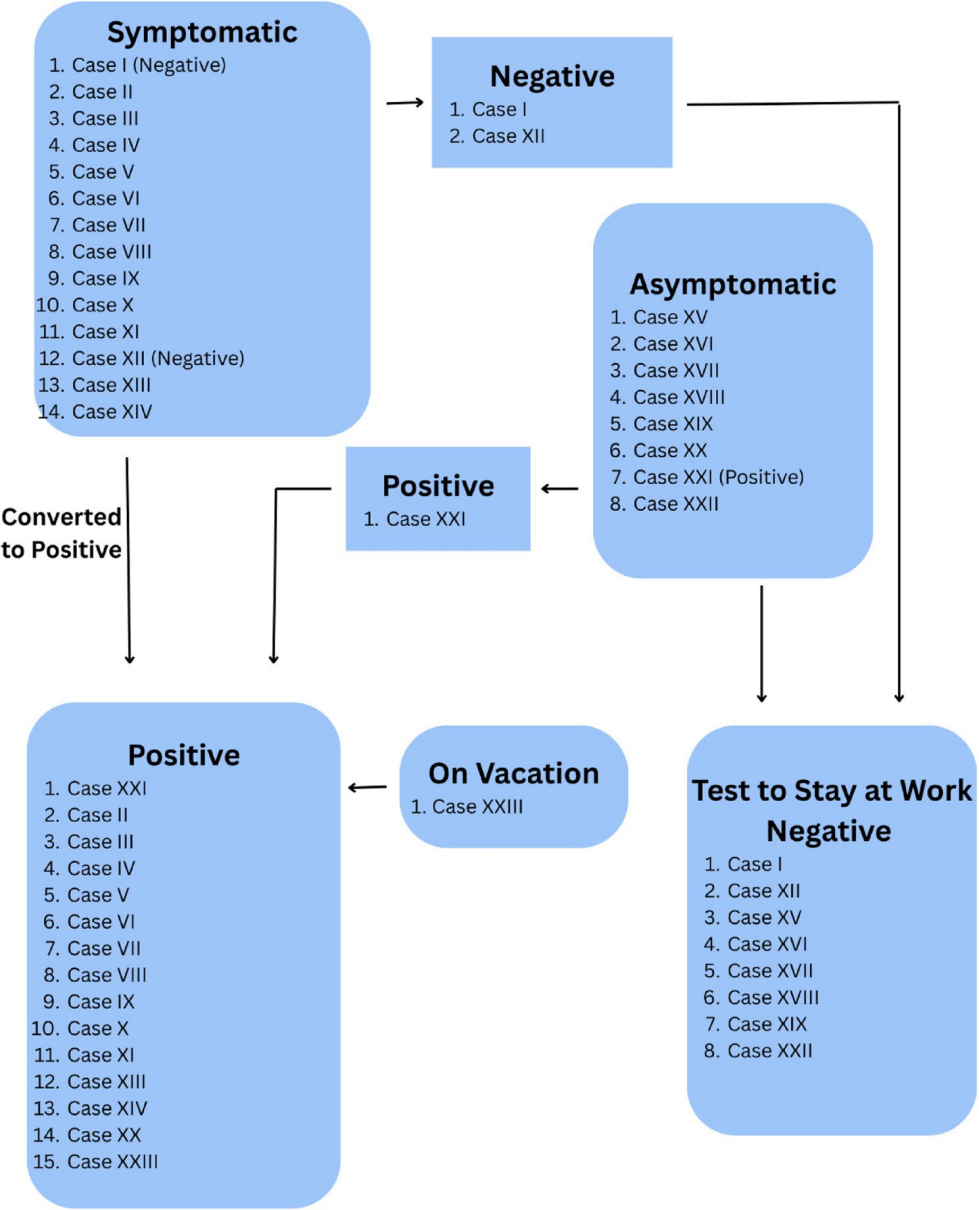
Outbreak 3

Outbreak 3 occurred in early August 2023. The outbreak is summarized schematically in Fig. 3. An offsite weekend-long team-building exercise brought together a total of 23 clinical HCP members (not counting non-HCP family members who also participated). The weekend event involved shared accommodations, meals, other group activities and long-distance carpooling for four hours or more. Symptom-based PCR (polymerase chain reaction) testing detected multiple cases and so surveillance testing of all attendees was conducted. In total, 15 of 23 HCP (65%) had confirmed COVID-19 infections within three days of the end of the retreat.

## Discussion

Consistent with the human resources policies of the SFVAHCS, nearly 100% of the HCP involved had received a primary COVID-19 vaccination series. The outbreaks we report all occurred in fairly rapid serial succession during the months of June through August 2023. Notably, there were no other outbreaks that occurred during this period and the examples did not reflect “selection” among a larger group of events. Although HCP occupational transmission might have occurred even with aggressive use of personal protection, maximal ventilation, and appropriate distancing, we did not experience other outbreaks of this magnitude absent these potential deficiencies, as noted. But we would not make the counter argument that the presence of such protective measures provides absolute assurance of successful control.

We did not perform genotyping which was difficult to obtain at our institution. This means that we cannot definitively link all of the infections through molecular biologic testing. The outbreaks, however, occurred telescoped in space and time, presumed to be due to the widely circulating COVID-19 strain in the community during the period in question.

The findings of our study support the view that nursing staff can be disproportionately impacted by work-related COVID-19 among HCP consistent with their frontline roles. This has been observed by others [10, 11]. This was more pronounced in Outbreak 3, however, where 15 out of 23 affected staff members were from the nursing service, because the staff who participated in the team-building exercise that led to the outbreak were from the nursing service only. Further, while Outbreak 3 was a work-related incident due to its alignment with efforts to enhance patient care services, it is important to emphasize that the outbreak occurred in a non-occupational setting.

The overall attack rate for the three outbreaks combined was 25% (95% CI: 18–32%). Although we calculated a combined attack rate for the outbreaks in order to provide an integrated public health context, this should not be misconstrued to imply homogeneity among the three events, particularly the scenario that was work-related but transpired outside the physical workspace for the purposes of team building. The three outbreak scenarios we describe may not be generalizable to health care facilities that adopted very different approaches to COVID-19 prevention among HCP during the epidemic phase and that also may undertake alternative control strategies in future infectious disease outbreaks.

Further, we cannot exclude all alternative factors that might have contributed to the outbreaks beyond lax masking, suboptimal ventilation, failure of HCP with symptoms to quickly self-quarantine, and, in the case of the offsite team building exercise, other unaccounted for external sources of contagion. In terms of sub-optimal ventilation specifically, even if this cannot be implicated with certainty, recommend guidelines for air exchanges and maintenance of filtering systems for health care facilities should be followed rigorously, even in satellite facilities.

These HCP outbreaks shed light on lingering vulnerabilities in our defenses against occupational COVID-19 [1–4]. This offers valuable lessons for bolstering infection control practices and preventing future transmission among HCP and their patients [12]. The liberal masking practices at the outpatient clinic sites likely facilitated viral spread [13]. There was indeed a lack of uniformity in masking policy, but there also was inconsistency in complying with whatever policy was current at the time. That is, some of the staff were not masking even where it was mandated to do so. Also, staff members were coming in to work despite having symptoms (some without masking) for which work absence until negative testing was mandated, failing to call the COVID hotline for guidance as required. There were no changes in staff interactions or space sharing introduced during the outbreaks, except for rapid activation of mandatory stricter masking for all out-patient clinic staff at all times and reinforcing a standard mandate that symptomatic staff not come to work until testing negative for COVID-19. The lack of additional physical distancing between HCP could represent a missed intervention. Additionally, ventilation systems in buildings could have contributed to spread at these sites [14]. Although the building leasing agents stated that air exchanges were appropriate to standards this could not be independently confirmed. In the absence of the verified air exchanges, the potential role of ventilation in COVID-19 transmission dynamics should not be discounted. The third outbreak provided ample opportunity for person-to-person spread including very high exposure scenarios such as group car travel and shared accommodations without masking.

Take-away lessons include rapid implementation of stricter masking as early as possible when an emerging outbreak is suspected, aggressive surveillance testing early in response to an outbreak in a discrete HCP working group and reinforcing education regarding not coming to work (or work events) when symptoms suggestive of COVID-19 are present [12, 13]. Finally, building ventilation may have contributed to two of the outbreaks and always needs to be checked periodically.

## Supplementary Information

Supplementary Material 1.

## Data Availability

No datasets were generated or analysed during the current study.
