# Supplementary figures and images for: Outbreaks of COVID-19 among healthcare personnel in a U. S. veterans administration health care system site, June and August 2023
Source: J Occup Med Toxicol. 2025 Aug 29;20:27. doi: 10.1186/s12995-025-00474-5 (PMC12398014; doi:10.1186/s12995-025-00474-5)

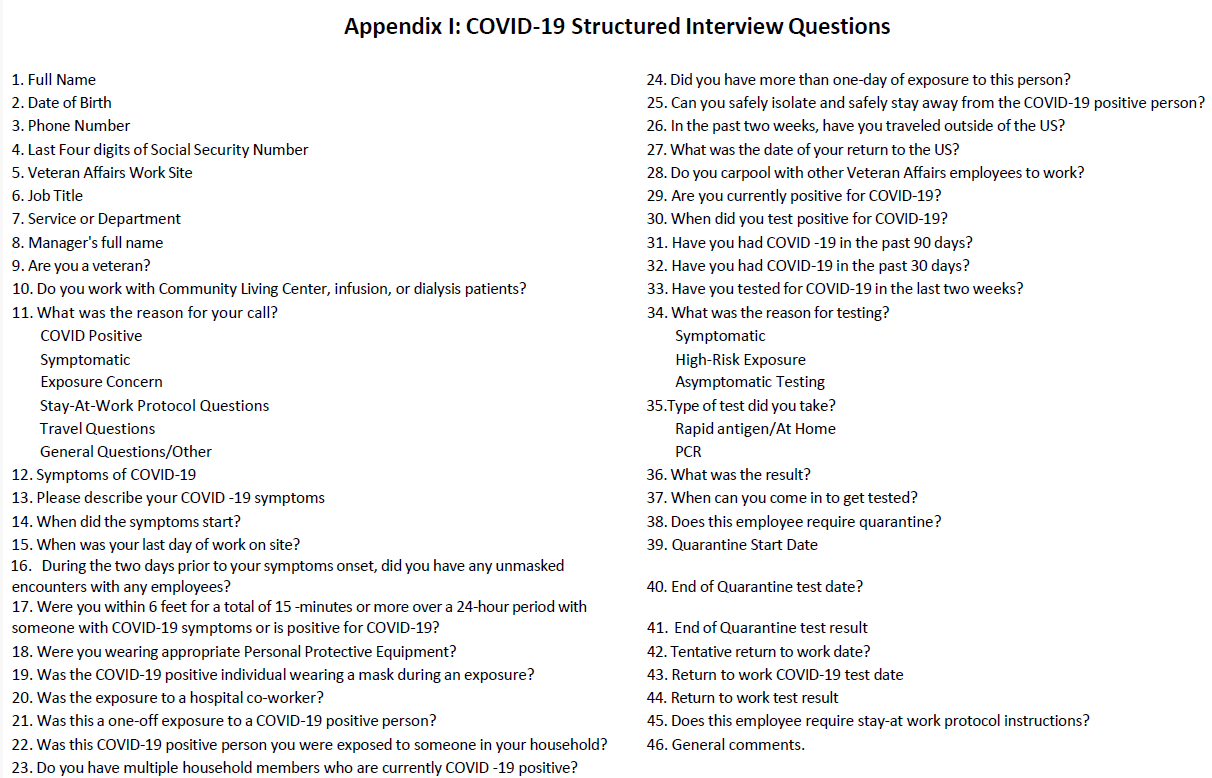

Supplement: Supplementary file 1 — Supplementary Material 1. [file 12995_2025_474_MOESM1_ESM.docx]
